# Slow Unfolding of Monomeric Proteins from Hyperthermophiles with Reversible Unfolding

**DOI:** 10.3390/ijms10031369

**Published:** 2009-03-24

**Authors:** Atsushi Mukaiyama, Kazufumi Takano

**Affiliations:** 1 Okazaki Institute for Integrative Bioscience, National Institutes of Natural Sciences, 5-1 Higashiyama, Myodaiji, Okazaki 444-8787, Japan; E-Mail: atsushi@ims.ac.jp; 2 Department of Material and Life Science, Osaka University, 2-1 Yamadaoka, Suita, Osaka 565-0871, Japan; 3 CREST, JST, 2-1 Yamadaoka, Suita, Osaka 565-0871, Japan

**Keywords:** Proteins from hyperthermophiles, folding/unfolding, stability, equilibrium and kinetic

## Abstract

Based on the differences in their optimal growth temperatures microorganisms can be classified into psychrophiles, mesophiles, thermophiles, and hyperthermophiles. Proteins from hyperthermophiles generally exhibit greater stability than those from other organisms. In this review, we collect data about the stability and folding of monomeric proteins from hyperthermophilies with reversible unfolding, from the equilibrium and kinetic aspects. The results indicate that slow unfolding is a general strategy by which proteins from hyperthermophiles adapt to higher temperatures. Hydrophobic interaction is one of the factors in the molecular mechanism of the slow unfolding of proteins from hyperthermophiles.

## 1. Introduction

Many organisms on Earth grow in extreme environments. Microorganisms whose optimal growth temperature is above 80 °C are called hyperthermophiles. Hyperthermophiles are found in hot environments such as deep-sea vents, submarine hydrothermal areas, and continental solfataras [1]. Most hyperthermophiles belong to archaea (e.g. *Thermococcus*, *Pyococcus*, and *Sulfolobus*), but some hyperthermophiles from bacteria (*Thermotoga* and *Aquifex*) have also been discovered.

Proteins from hyperthermophiles usually exhibit higher stability than those from organisms that grow at lower temperatures, so it is expected that studies of proteins from hyperthermophiles will provide general or additional insights into the forces stabilizing the native conformation of proteins [2]. In recent years, many genomes from hyperthermophiles have been sequenced, and a number of the crystal structures of proteins from hyperthermophiles have been determined. This enables us to compare the sequences and the crystal structures of homologous proteins between hyperthermophiles and other organisms growing in moderate temperatures. These comparative studies indicate that the higher stability of proteins from hyperthermophiles is associated with several factors such as increased salt bridges, improved hydrogen bonding, favorable packing interactions, fewer cavities, and improved hydrophobic interactions [3–7]. It has been suggested that proteins from hyperthermophiles use various combinations of these stabilizing factors.

To elucidate the stabilization mechanism of proteins from hyperthermophiles, a thermodynamic analysis is also useful [8–11]. Many studies concerning the thermodynamic stability of proteins from hyperthermophiles focus primarily on the equilibrium aspects, which reveal that extremely high stability can be achieved in these proteins by increasing the number of ionic interactions and the extent of hydrophobic surface burial [12–14]. Furthermore, it has recently been reported that the stability of some proteins is under kinetic control *in vivo* and *in vitro* [15–22]. Luke *et al*. [23] summarized the data on thermodynamic and kinetic folding behavior of proteins from hyperthermophiles and found that the unfolding is slower for proteins from hyperthermophiles than for the mesostable ones. The data, however, includes data from oligomeric or monomeric proteins with irreversible or multi-state folding.

This review focuses on studies of monomeric proteins from hyperthermophiles with reversible unfolding, examined from both equilibrium and kinetic aspects. This will provide an essential understanding of stabilization linked to the slow unfolding of proteins from hyperthermophiles. In the following section, we describe how the stability and folding of proteins from hyperthermophiles are experimentally characterized by thermodynamic and kinetic analyses. Next, we report on our studies of the stability and folding of ribonuclease HII from a hyperthermophilic archaeon, *Thermococcus kodakaraensis* (Tk-RNase HII) and introduce other studies of proteins from hyperthermophiles. We conclude that slower unfolding is a fundamental characteristic of the architectural principles of proteins from hyperthermophiles. Moreover, we discuss the molecular mechanism of the slow unfolding of proteins from hyperthermophiles.

## 2. Characterizing the Stability and Folding of Hyperthermophilic Proteins

The stability of proteins in solution is evaluated quantitatively by Gibbs energy changes (ΔG) observed upon unfolding, when the reaction is reversible under experimental conditions. ΔG is obtained experimentally by perturbing the native state using temperature and a denaturant (e.g., urea or guanidine hydrochloride (GdnHCl)) and monitoring the shift in the equilibrium by various biophysical techniques such as differential scanning calorimetry (DSC), fluorescence, and circular dichroism (CD) [24]. Assuming a simple two-state process (Native, N ⇌ Unfolded, U), ΔG is given by:

(1) ΔG=− RTlnK,K=[U]/[N]

where R, T, and *K* represent the gas constant, temperature, and equilibrium constant. The temperature dependence of ΔG (stability profile) provides more valuable information about the thermodynamic stability of proteins. The temperature dependence of ΔG is expressed as:

(2) $$\Delta \text{G}(\text{T})=\Delta {\text{H}}_{\text{m}}-\text{T}\;\Delta {\text{H}}_{\text{m}}/{\text{T}}_{\text{m}}+\Delta {\text{C}}_{\text{p}}[\text{T}-{\text{T}}_{\text{m}}-\text{T}\;\text{ln}\;(\text{T}/{\text{T}}_{\text{m}})]$$

where ΔH_m_ is the enthalpy of unfolding at the transition midpoint temperature (denaturation temperature: T_m_) and ΔC_p_ is the difference in heat capacity between the native and unfolded states.

**Figure 1.**
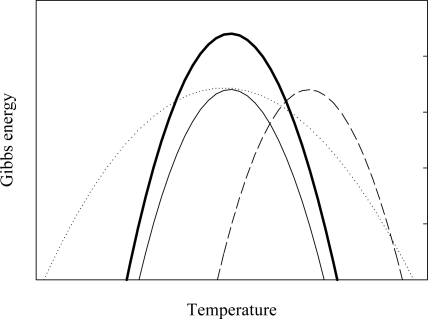
Stability profiles depicting the different ways to increase the denaturation temperature (T_m_). The solid thin line represents the stability curve for a hypothetical protein from an organism growing in moderate temperature. Proteins from hyperthermophiles can adapt to higher temperature by (i) shifting the curve up (solid thick line), (ii) shifting the curve to the right (dashed line), and (iii) flattening the curve (dotted line).

Three models are proposed for proteins from hyperthermophiles to adapt to higher temperatures compared with those from organisms growing in moderate temperatures: Proteins from hyperthermophiles (i) raises the entire stability curve to a higher ΔG, (ii) shifts the stability curve towards higher temperatures, and (iii) reduces ΔC_p_, resulting in a flatter stability curve (Figure 1).

All three models are observed in nature and, in many cases, proteins from hyperthermophiles do not utilize a single mechanism but rather various combinations of (i) to (iii) [25–31]. It has been experimentally shown that the core hydrophobicity and electrostatic interaction at the surface in proteins from hyperthermophiles are involved in the temperature dependence of ΔG [13,30]. For example, ribosomal protein L30e from a hyperthermophile, *Thermococcus celer*, adapts to higher temperatures by utilizing models (i) and (iii) compared with its mesophilic homologue from yeast and the mutational study reveals that electrostatic interactions contribute to the greater stability and reduced ΔC_p_ of L30e from *Thermococcus celer* [30].

ΔG is also determined by kinetic unfolding/folding experiments. In kinetic experiments, a perturbation is imposed by changing the denaturant concentration, pH, or temperature. The time course of the reaction is then monitored by the various biophysical techniques described above [32]. Assuming a protein that displays a simple two-state transition, the reactions of unfolding and refolding curves are fit to a single exponential, yielding a single apparent rate constant *k*_app_ where *k*_app_ = *k*_unf_ + *k*_ref_, and *k*_unf_ and *k*_ref_ represent the unfolding and refolding rate constants. For the unfolding and refolding kinetics of a denaturation concentration jump, the logarithm of the apparent rate constant (*k*_app_) of the unfolding and refolding linearly depends on the final denaturant concentration. The dependence of ln *k*_app_ on the denaturant concentration is generally analyzed using the following equation:

(3) $$\text{ln}\;k_{\text{app}}=\text{ln}\;\{k_{\text{ref}}({\text{H}}_{2}\text{O})\;\text{exp}\;(-m_{\text{ref}}[\text{D}])+k_{\text{unf}}({\text{H}}_{2}\text{O})\;\text{exp}\;(+m_{\text{unf}}[\text{D}])\}$$

Here, *k*_unf_(H_2_O) and *k*_ref_(H_2_O) represent the unfolding and refolding rate constants in the absence of denaturant and *m*_unf_ and *m*_ref_ are their dependencies on the denaturant concentration. [D] is the final denaturant concentration. Importantly, ΔG is also obtained using the rate constants of unfolding and refolding reactions. For a simple two-state transition, the equilibrium constant (*K*) is expressed as the ratio of the refolding rate constant to the unfolding one (*K* = *k*_f_/*k*_u_).

**Figure 2.**
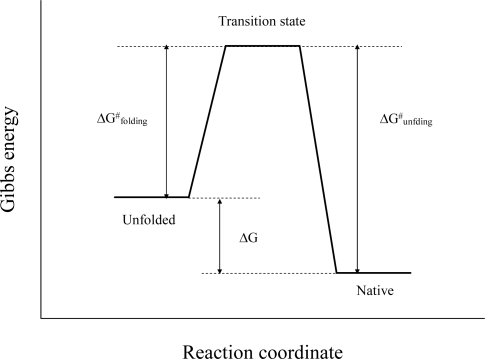
Schematic representation of the energy diagram for the protein folding process, assuming a two-state model. ΔG^#^_folding_ and ΔG^#^_unfolding_ denote the activation energy between the unfolded and transition states and between the respective native and transition states. The activation energy can be determined from Eyring’s equation.

Figure 2 displays a Gibbs energy diagram of the folding pathway for protein, assuming a two-state model. Hence, the increased stability of proteins from hyperthermophiles is caused by the slower unfolding rate (the larger ΔG^#^_unfolding_ value), the faster refolding rate (the smaller ΔG^#^_folding_ value), or both.

## 3. Ribonuclease HII from *Thermococcus kodakaraensis*

**Figure 3.**
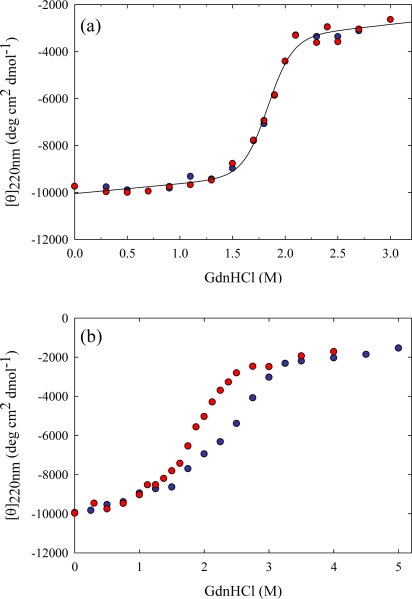
GdnHCl-induced unfolding (blue circles) and refolding (red circles) curves of Tk-RNase HII at (a) 50 °C for two weeks and (b) 20 °C for one month, monitored by measuring CD at 220nm. The solid line in (a) is the best fit to a two-state equation. For unfolding, Tk-RNase HII was incubated in GdnHCl at different concentrations. For refolding, the protein, which was unfolded completely at a 4M GdnHCl concentration, was diluted with buffer and the diluted protein solution was incubated. Figures are reproduced from [20].

Tk-RNase HII is a monomeric protein consisting of 228 amino acid residues whose stability and folding are well characterized by our research [20,33–36]. RNase H hydrolyzes only the RNA strand of an RNA/DNA hybrid [37]. The enzyme is ubiquitously present in various organisms and is involved in DNA replication and repair [38]. The crystal structures of Tk-RNase HII and its several variants have been determined [39–41]. The protein contains an α/β fold, known as the RNase H-fold due to its characterization in *Escherichia coli* RNase HI. Tk-RNase HII exhibits high reversibility against both heat- and GdnHCl-induced unfolding. Equilibrium unfolding by GdnHCl is a two-state process, with a reaction at 50 °C attaining equilibrium in two weeks (Figure 3a). ΔG in water (ΔG(H_2_O)) at 50 °C, obtained from the equilibrium unfolding curve, is 43.6kJ mol^−1^, indicating that this protein is very stable at 50 °C compared to mesophilic proteins.

The dependence of ΔG on the GdnHCl concentration (*m* value) is 23.6kJ mol^−1^ M^−1^. However, the unfolding curve is inconsistent with the refolding curve at 20 °C even after 30 days (Figure 3b). The inconsistency between the refolding and unfolding curves is due to the remarkably slow unfolding of Tk-RNase HII. It takes about two months to reach equilibrium at 20 °C.

**Figure 4.**
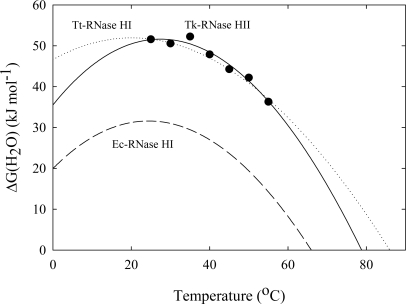
Thermodynamic stability profiles of Tk-RNase HII, Ec-RNase HI, and Tt-RNase HI, obtained from GdnHCl-induced equilibrium unfolding experiments [42]. The solid, dashed, and dotted lines represent Tk-RNase HII, Ec-RNase HI, and Tt-RNase HI. Figure is reproduced from [20].

The dependence of ΔG(H_2_O) on the temperature (stability profile) for Tk-RNase HII was investigated and compared with that for the mesophilic bacterium *Escherichia coli* (Ec-RNase HI) and the thermophilic bacterium *Thermus thermophilus* (Tt-RNase HI) [42]. Figure 4 plots the stability profiles for the three proteins. Tt-RNase HI increases the stability by shifting the stability curve up and flattening it compared with Ec-RNase HI. The stability profile for Tk-RNase HII displays a maximum around 40 °C, which is higher than that of Ec-RNase HI and Tt-RNase HI. The ΔC_p_ of Tk-RNase HII, 14.5kJ mol^−1^ K^−1^, exceeds that of Tt-RNase HI. Therefore, Tk-RNase HII adapts to higher temperature by shifting the stability curve up and to the right. The results suggest that the stabilization mechanisms of Tk-RNase HII and Tt-RNase HI are different.

**Figure 5.**
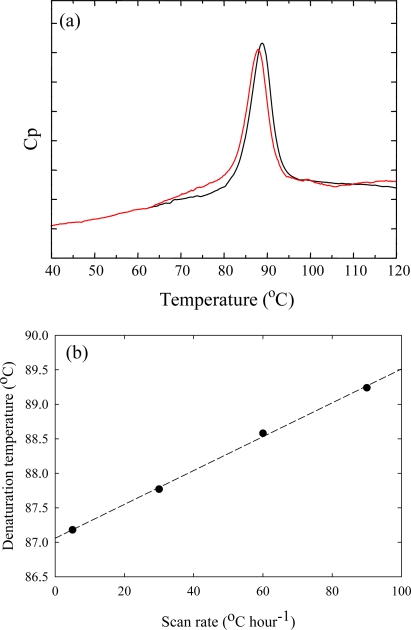
(a) Dependence of DSC curves of Tk-RNase HII on the scan rate. Samples with the same protein concentration were measured at different scan rates. Red and black lines represent the DSC curves at scan rates of 30 and 60 °C hour^−1^. (b) Dependence of the denaturation temperature on scan rate. The dashed line represents a linear fit. Figures are reproduced from [20].

Heat-induced unfolding experiments with Tk-RNase HII were carried out by DSC. The results demonstrate the high stability and slow unfolding of Tk-RNase HII. Figure 5(a) presents DSC curves for Tk-RNase HII at scan rates of 30 and 60 °C hour^−1^. The denaturation temperature in the DSC curve for Tk-RNase HII shifts as a function of the scan rate. The denaturation temperature at a scan rate of 90 °C hour^−1^ is 89.2 °C, whereas the denaturation temperature at 5 °C hour^−1^ is 87.2 °C. This indicates that the heat-induced unfolding of Tk-RNase HII does not attain equilibrium at these scan rates because of the remarkably slow unfolding. Heat-induced unfolding of most mesophilic proteins can attain equilibrium at 60 °C hour^−1^. Figure 5(b) plots the dependence of the denaturation temperature as a function of the scan rate. The extrapolated value is 87.1 °C. Although the linear fit is not theoretical, this value indicates that Tk-RNase HII is very stable against heat-induced denaturation.

**Figure 6.**
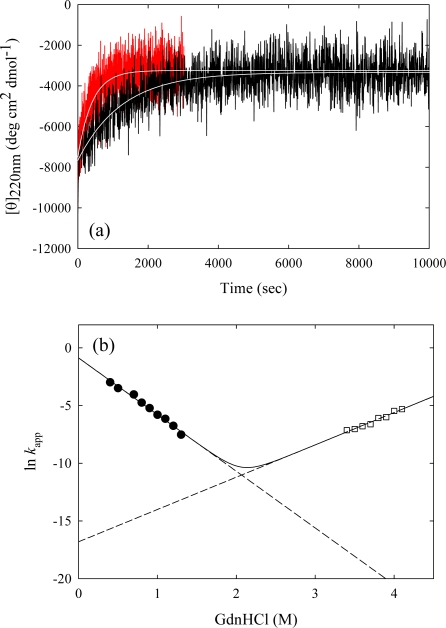
(a) Representative kinetic unfolding curves for Tk-RNase HII at 50 °C, monitored by a change in CD at 220nm. The reaction was initiated by GdnHCl concentration jumps to 3.9M (red line) and 3.4M (black line). Unfolding curves are fit to a single exponential. (b) GdnHCl concentration dependence of the apparent rate constants (*k*_app_) of the unfolding (open squares) and refolding (closed circles) kinetics of Tk-RNase HII at 50 °C. The solid line represents the theoretical curve using Equation (3). Figures are reproduced from [20].

The unfolding and refolding kinetics by GdnHCl concentration jump was examined at 50 °C. Figure 6(a) shows the typical unfolding curves of Tk-RNase HII. The observed kinetics of unfolding and refolding reaction is fit to a single exponential. Figure 6(b) shows the dependence of the logarithm of the apparent rate constant of the unfolding and refolding on the final GdnHCl concentration. The *k*_unf_(H_2_O) and *k*_ref_(H_2_O) at 50 °C are 5.0 × 10^−8^ s^−1^ and 7.8 × 10^−1^ s^−1^, and *m*_unf_ and *m*_ref_ at 50 °C are 2.8 M^−1^ s^−1^ (7.5 kJ mol^−1^ M^−1^) and 5.5 M^−1^ s^−1^ (14.8 kJ mol^−1^ M^−1^), respectively. The ΔG(H_2_O) value obtained from those rate constants using a two-state model at 50 °C, 44.5 kJ mol^−1^, is coincident with that from the equilibrium study, 43.6 kJ mol^−1^. This suggests two-state folding/unfolding of Tk-RNase HII. The fractional change of the *m* value during refolding α, where β = *m*_ref_ / (*m*_ref_ - *m*_unf_), yields 0.66, indicating that the transition state of folding/unfolding is similar with the native state rather than the unfolded state in its solvent accessible area [43].

The kinetics of the folding and refolding reactions of Ec-RNase HII and Tt-RNase HI have been studied in detail at 25 °C [44,45]. To compare the unfolding and refolding rate constants for Tk-RNase HII with those for Ec-RNase HII and Tt-RNase HI, the kinetic experiments were also carried out at 25 °C. The unfolding rate constant for Tk-RNase HII at 25 °C (6.0 × 10^−10^s^−1^) is much smaller than those for Ec-RNase HI (1.1 × 10^−5^s^−1^) and Tt-RNase HI (4.0 × 10^−6^s^−1^). In contrast, little difference is observed among these proteins in the refolding rate constant for the formation of the native state. These results indicate that the stabilization of Tk-RNase HII originates from the remarkably slow unfolding rate.

## 4. Other Proteins

### 4.1. Cold shock protein (Csp)

The stability and folding of cold shock proteins from *Thermotoga maritima* (hyperthermophilic bacterium, Tm-Csp), *Bacillus caldolyticus* (thermophilic bacterium, Bc-Csp), and *Bacillus subtilis* (mesophilic bacterium, Bs-Csp) have been investigated in detail [46]. These proteins are small (66 to 68 amino acid residues) and adopt all-0 sheet folds in their native structures. GdnHCl-induced equilibrium unfolding is a two-state process, and the kinetic intermediates are not accumulated during the unfolding and refolding reactions (20 °C). Tm-Csp exhibits higher stability (26.2kJ mol^−1^) than Bc-Csp (20.1kJ mol^−1^) and Bs-Csp (11.3kJ mol^−1^). Coulombic interactions at the surface unique to Tm-Csp are involved in an increase in the stability [47]. The fractional change of the *m* value during folding/unfolding (α value) was 0.86 for Tm-Csp and 0.93 for Bc-Csp, indicating that the transition states of refolding of all cold shock proteins are almost native-like in their interactions with the solvent. The unfolding rate constant of Tm-Csp is one order of magnitude smaller than that of Bc-Csp and two orders of magnitude smaller than that of Bs-Csp, while the refolding rate constants are similar for the three proteins. An increase in the equilibrium stability of Tm-Csp is brought about by the slower unfolding. The slower unfolding for Tm-Csp relative to Bs-Csp is observed over a wide temperature range. The difference is found to be due to a difference in the activation entropy of unfolding [48].

### 4.2. Ribosomal protein S16

The folding thermodynamics and kinetics of ribosomal protein S16 from the hyperthermophilic bacterium *Aquifex aerolicus* (S16_thermo_) have been investigated and compared with those of ribosomal protein S16 from the mesophilic bacterium *Chlamydia pneumoniae* (S16_meso_) [49]. S16_thermo_ consists of 112 amino acid residues, and its crystal structure has been determined. Thermal unfolding experiments demonstrate that the T_m_ values for S16_thermo_ and S16_meso_ are 111 °C and 59 ° C. Equilibrium unfolding by urea of S16_thermo_ and S16_meso_ is a two-state process. The stability profile of S16_thermo_ is shifted up and flattened, resulting in a reduced AC_p_, and shifted toward higher temperatures than that of S16_meso_. The unfolded state of S16_thermo_ is more compact relative to S16_meso_, suggesting that residual structures in the unfolded state of S16_thermo_ are involved in the increased stability. The unfolding rate constant for S16_meso_ is two orders of magnitude smaller than that of S16_meso_, indicating that the difference in unfolding rate accounts for the difference in stability between the two proteins. For refolding, the rate constants for the formation of the native state are similar for both proteins.

## 5. Molecular Mechanisms of the Slow Unfolding of Hyperthermophilic Proteins

From the data presented above, slower unfolding seems to be a common characteristic of proteins from hyperthermophiles. However, the molecular basis for the slow unfolding of proteins from hyperthermophiles remains elusive. Here, we describe our recent studies in which the contributions of various stabilization factors to the slower unfolding of Tk-RNase HII are investigated. Moreover, we propose a novel viewpoint for future work concerning the evolutionary background of the slow unfolding of proteins from hyperthermophiles.

### 5.1. Hydrophobic interactions

**Figure 7.**
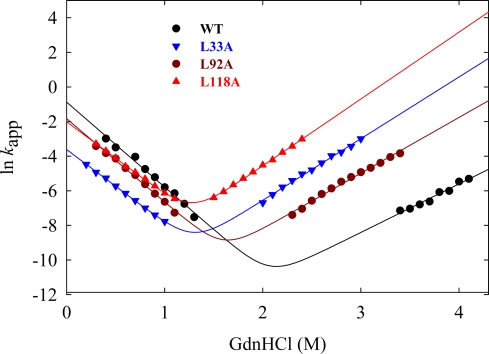
GdnHCl concentration dependence of the logarithm of the apparent rate constant (*k*_app_) for the unfolding and refolding kinetics of the wild-type and three mutant proteins of Tk-RNase HII at 50 °C. The solid line represents the theoretical curve using Equation (3). Figure is reproduced from [34].

**Figure 8.**
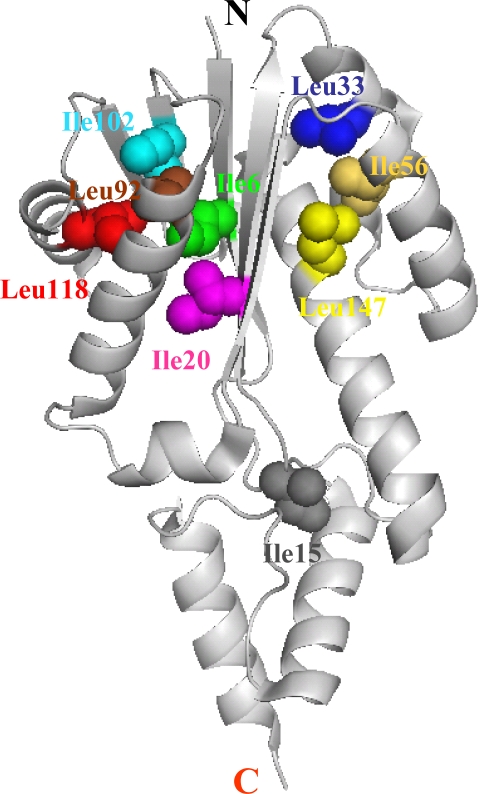
Crystal structure of Tk-RNase HII, depicting the side chains of Ile and Leu that have been substituted. Figure is reproduced from [34].

The hydrophobic effect is one of the important stabilizing forces of folded proteins [50–54]. Systematic mutational analysis reveals that buried hydrophobic residues contribute to the extremely slow unfolding of Tk-RNase HII [34]. Mutant proteins in which a larger hydrophobic side chain is replaced by a smaller one (Lue/Ile to Ala) are destabilized by 8.9 to 22.0 kJ mol^−1^ at 50 °C. The unfolding rate constants of these mutant proteins are one to three orders of magnitude greater than that of the wild-type protein (Figure 7). These mutant proteins refold more slowly, but the difference is less than one order of magnitude. The mutation sites are completely buried, with their locations distributed widely throughout the molecule (Figure 8). These results indicate that buried hydrophobic residues strongly contribute to the kinetic robustness of Tk-RNase HII. This is the first report that presents a practical cause for the slow unfolding of proteins from hyperthermophiles. In contrast, it has been reported from simulation studies that protein-water interactions at protein surface rather than internal packing are important for the stabilization of proteins from hyperthermophiles [56,57]. Today, it remains unclear whether protein-water interactions at the surface contribute to the slow unfolding of proteins from hyperthermophiles, and it is an interesting subject which should be solved.

### 5.2. The Proline Effect

Proline residues decrease the conformational entropy of the denatured state and consequently lead to protein stabilization [57]. In particular, it has been reported that proline residues introduced at N-terminal α-helices increase the thermostability of proteins [58]. From sequence comparisons among homologous proteins with different thermostability, proline residues are more ubiquitous at α-helix N-terminals in more stable proteins [59]. Hence, the effects of proline residues at the N-terminal α-helices on the stability and slow unfolding of Tk-RNase HII have been tested by mutational analysis [35]. The results indicate that Tk-RNase HII is stabilized by proline residues at the N-terminal α-helices. In contrast, the unfolding rate for the mutant proteins changes less than one order of magnitude compared with that of the wild-type protein, indicating that proline residues contribute little to the slow unfolding of Tk-RNase HII.

### 5.3. The osmolyte effect

The effects of osmolyte on the stability and folding of Tk-RNase HII have been investigated [36]. Osmolytes are small molecules that protect plants, animals and microorganisms, including hyperthermophiles, from harsh environmental stress [60,61]. The presence of the osmolyte trimethyl-amine-*N*-oxide (TMAO) in solution increases the stability of Tk-RNase HII. Kinetic experiments indicate that the *k*_u_(H_2_O) value in the presence of 0.5M TMAO is about one-seventh of that in the absence of TMAO, and that the *k*_r_(H_2_O) value in the presence of 0.5M TMAO is about seven times higher than that in the absence of TMAO, indicating that TMAO affects the unfolding and refolding kinetics of Tk-RNase HII to a similar extent. Since similar effects of osmolytes on unfolding and refolding rates have been observed in several proteins from humans and mesophiles [62,63], a decrease in unfolding rate by osmolytes is not intrinsic for proteins from hyperthermophiles.

### 5.4. Evolutionary background of the slow unfolding of hyperthermophilic proteins

The degree of the decrease in unfolding rate of proteins from hyperthermophiles relative to their mesophilic counterparts varies, depending on the protein. For example, the unfolding rate constant for Tk-RNase HII is much less (by five orders of magnitude) than that for its mesophilic counterpart. By contrast, the unfolding rate constant for Tm-Csp and S16_thermo_ is slightly smaller (by two orders of magnitude) than that of its mesophilic homolog. The origin of such a difference remains obscure at this point. Here, we propose one possibility for this discrimination.

*Thermococcus kodakaraensis* belongs to archaea [64], but *Thermotoga maritima* and *Aquifex aeolicus* are bacteria [65,66]. Archaea and bacteria divided at an early stage of the evolutionary tree [67,68]. It has been proposed that their proteins have different stabilization mechanisms [69]. Proteins from archaea that originated in a hot environment are more compact and hydrophobic than their mesophilic homologs. In contrast, proteins from some bacteria that later recolonized under extreme conditions are stabilized by specific interactions such as salt bridges. The difference in the unfolding characteristics of proteins from hyperthermophiles may be ascribed to this difference in stabilization mechanism between archaea and bacteria. The strong effect of hydrophobic interaction on the slow unfolding of Tk-RNase HII supports this hypothesis. We will confirm this in the future.

## 6. Conclusions

Previous studies showed that the unfolding is slower for proteins from hyperthermophiles than for the mesostable ones, as summarized in the review by Luke *et al*. [23]. The results, however, includes data from oligomer or monomer proteins with irreversible or multi-state folding. In this review, we focus on monomeric proteins from hyperthermophiles with reversible unfolding examined in terms of the equilibrium and kinetic aspects. Unfortunately, the number of studies is quite low. Table 1 provides thermodynamic and kinetic data for proteins from hyperthermophiles described in this review. More studies are required to clarify the universal mechanism of the stabilization of proteins from hyperthermophiles. However, we found a clear trend whereby the stabilization mechanism of proteins from hyperthermophiles originates from slow unfolding, based on data from monomeric proteins with reversible unfolding. This means that the slow unfolding is a basic characteristic of the architectural principles of proteins from hyperthermophiles, because other factors such as oligomerization and uncertainties due to irreversible reactions can be eliminated.

## Figures and Tables

**Table 1.** Thermodynamic and kinetic parameters characterizing the stability and refolding/unfolding of proteins from hyperthermophiles.

Protein	Organism	G(H_2_O) (kJ mol^−1^)	*k*_ref_(H_2_O) (s^−1^)	*k*_ref_(H_2_O) (s^−1^)
RNase HII	*Thermococcus*	43.6 (50 °C)	5.0 × 10^−8^ (50°C)	7.8 × 10^−1^ (50 °C)
	*kodakaraensis*	48.3 (25 °C)	6.0 × 10^−10^ (25 °C)	0.4 × 10^−1^ (25 °C)
Cold shock protein	*Thermotoga maritima*	26.2 (25 °C)	1.8 × 10^−2^ (25 °C)	5.7 × 10^2^ (25 °C)
Ribosomal protein S16	*Aquifex aeolicus*	24.8 (25 °C)	1.6 × 10^−2^ (25 °C)	3.8 × 10 (25 °C)
